# Perioperative Application of Levosimendan Optimizes Postoperative Renal Function and Organ Perfusion in Patients with Severe Heart Failure

**DOI:** 10.3390/jcdd10070312

**Published:** 2023-07-20

**Authors:** Vasileios Leivaditis, Manfred Dahm, Anastasia Papaporfyriou, Michail Galanis, Efstratios Koletsis, Nikolaos Charokopos, Benjamin Ehle, Athanasios Papatriantafyllou, Erich Haussmann, Vladislav Kaplunov, Konstantinos Grapatsas

**Affiliations:** 1 Department of Cardiothoracic and Vascular Surgery, Westpfalz-Klinikum, 67655 Kaiserslautern, Germany; mdahm@westpfalz-klinikum.de (M.D.); ehaussmann@westpfalz-klinikum.de (E.H.); vkaplunov@westpfalz-klinikum.de (V.K.); 2 Department of Pulmonology, Internal Medicine II, Vienna University Hospital, 1090 Vienna, Austria; anastasia.papaporfyriou@meduniwien.ac.at; 3 Department of Thoracic Surgery, Inselspital, Bern University Hospital, University of Bern, 3012 Bern, Switzerland; michail.galanis@insel.ch; 4 Department of Cardiothoracic Surgery, University Hospital of Patras, 26504 Patras, Greece; ekoletsis@hotmail.com (E.K.); nc.7@hotmail.com (N.C.); 5 Department of Thoracic Surgery, Asklepios Lung Clinic Munich-Gauting, 82131 Gauting, Germany; benjaminehle84@gmail.com; 6 Department of General Surgery, General Hospital of Patras “Agios Andreas”, 26332 Patras, Greece; thanospap9@yahoo.gr; 7 West German Lung Center, Department of Thoracic Surgery and Thoracic Endoscopy, Ruhrlandklinik, University Hospital Essen, University Duisburg-Essen, 45239 Essen, Germany; grapatsaskostas@gmail.com

**Keywords:** levosimendan, heart failure, cardiac surgery, renal insufficiency, cardiac injury, organ and tissue perfusion, systematic inflammatory response, metabolic balance

## Abstract

*Background:* Renal dysfunction and impaired organ perfusion are common concerns following cardiac surgery. Levosimendan, a calcium sensitizer inotropic drug, is investigated in this study for its potential to improve postoperative renal function and organ perfusion in patients with low preoperative ejection fraction and severe myocardial dysfunction after cardiac surgery. *Methods:* A retrospective analysis was conducted on 314 patients with preoperative heart failure who underwent cardiac surgery. Among them, 184 patients received perioperative adjunctive therapy with levosimendan, while 130 patients with similar characteristics received conventional treatment. *Results:* The perioperative administration of levosimendan resulted in a significantly lower need for renal replacement therapy (*p* < 0.001) and improvements in the serum creatinine levels, glomerular filtration rate, and creatinine clearance. Similarly, the C-reactive protein levels, blood pH, and lactic acid levels showed comparable improvements. *Conclusions:* The use of levosimendan was associated with a significant enhancement in postoperative renal function and a reduction in the need for renal replacement therapy. Furthermore, it resulted in a decrease in the extent of organ malperfusion. Postoperative inflammatory reactions and metabolic balance also exhibited improvements.

## 1. Introduction

Heart failure is a widespread and debilitating condition with a significant global impact. Cardiac surgery is frequently necessary for patients with severe heart failure, but it carries a high risk of peri- and postoperative complications. One of the most common complications is postoperative organ dysfunction, particularly renal dysfunction. Organ protection during the perioperative period plays a crucial role in determining postoperative outcomes. Postoperative renal dysfunction following cardiac surgery is a prevalent complication associated with a mortality rate of 60–90% and prolonged stays in the intensive care unit (ICU) and hospital. Its incidence has been reported to reach up to 55% [1,2].

Renal failure, defined by a glomerular filtration rate (GFR) < 60 mL/min/1.73 m^2^ in patients with heart failure, is linked to significantly higher mortality compared with heart failure patients without renal insufficiency [3]. Additionally, acute kidney injury (AKI) often leads to the development of chronic kidney disease [4,5]. The interplay between cardiac and renal dysfunction in managing heart failure has given rise to the concept of cardiorenal syndrome (CRS) [6]. CRS refers to the pathophysiologic interaction between the heart and kidneys, where acute or chronic impairment of either organ’s function can result in the dysfunction of the other [6,7].

The etiology of acute kidney injury after cardiac surgery is multifactorial. Risk factors for renal function decline include a history of chronic renal failure, acute coronary syndrome, arterial hypertension, diabetes mellitus, advanced age, and severe atherosclerosis [8]. Additionally, factors such as the inflammatory response, oxidative stress, transient hemodynamic derangements, and ischemia/reperfusion injury have been implicated. Open-heart surgery triggers a systemic inflammatory response syndrome (SIRS) and affects organ perfusion, which can alter the metabolic balance of patients postoperatively. These perioperative changes may lead to severe complications [9,10].

Levosimendan, a calcium sensitizer, enhances myocardial contractility by increasing the sensitivity of troponin C to calcium. It also possesses vasodilatory properties, reducing afterload and preload. These effects improve cardiac function and increase cardiac output. This study aims to investigate the perioperative effects of levosimendan administration on postoperative renal function, systemic inflammatory response, and the metabolic balance of patients.

## 2. Materials and Methods

### 2.1. Study Design

This retrospective, single-center study analyzed 314 patients with preoperative heart failure who underwent cardiac surgery. The patients were divided into two groups: a levosimendan group and a control group, based on the perioperative administration of levosimendan. Data from patients who received levosimendan were compared with a historical group of patients who underwent similar operative procedures but did not receive the drug. The inclusion of patients in the historical group was based on ensuring comparability within a similar time frame, considering the inherent limitations of retrospective studies. Although patient allocation was not randomized, it was random and comparable within the context of this retrospective analysis.

### 2.2. Definition of Renal Failure

We based our staging of renal failure on established clinical guidelines from reputable organizations, such as the National Kidney Foundation (NKF) and the Kidney Disease: Improving Global Outcomes (KDIGO) group. These guidelines consider essential parameters, including GFR, urine albumin-to-creatinine ratio (ACR), and the presence of kidney damage, to classify renal failure into distinct stages. The staging system divides renal failure into five stages.

- Stage 1: mild kidney damage and normal or near-normal GFR (>90 mL/min/1.73 m^2^);
- Stage 2: mild to moderate kidney damage and a slightly reduced GFR (60–89 mL/min/1.73 m^2^);
- Stage 3:
  ○ Stage 3A: moderate reduction in GFR (45–59 mL/min/1.73 m^2^);
  ○ Stage 3B: severe reduction in GFR (30–44 mL/min/1.73 m^2^);
- Stage 4: severe kidney damage and a significant decline in GFR (15–29 mL/min/1.73 m^2^);
- Stage 5: end-stage renal, GFR is severely reduced (<15 mL/min/1.73 m^2^) or dialysis is required.

### 2.3. Surgical Procedure, Postoperative Course, and Application of Levosimendan

The operations took place at the Department of Cardiothoracic and Vascular Surgery of Westpfalz–Klinikum Hospital in Kaiserslautern, Germany, from 2008 to 2017. The surgical procedures included coronary artery bypass graft surgery (CABG), heart valve surgeries, and combinations of these procedures, both on a regular and emergency basis. Patient treatment followed the current guidelines of the European Society of Cardiology (ESC) and the European Association of Cardiothoracic Surgery (EACTS). All patients were postoperatively transferred to and treated in the ICU. Subsequently, they were moved to the intermediate care unit and then to the regular ward for further treatment, regardless of whether they received levosimendan or not. Patients who received levosimendan were provided thorough information about its perioperative use and gave their consent prior to the operation. Levosimendan, in the form of Simdax^®^, was administered at a dose of 0.1 μg/kg/min, hemodynamically tolerated, via both central venous and peripheral venous accesses. For patients with preexisting renal and hepatic impairment, appropriate monitoring of renal and hepatic function was conducted. Hemodynamic monitoring in the ICU guided the administration of levosimendan. In cases of persistent hypotension, despite additional intravenous volumes and norepinephrine support, levosimendan was discontinued, and those patients were excluded from the study.

### 2.4. Patients’ Follow-Up

The patients’ postoperative follow-up was conducted at the Department of Cardiothoracic and Vascular Surgery of Westpfalz–Klinikum Hospital in Kaiserslautern. Additionally, follow-up appointments were scheduled at the Department for Cardiology, as well as through the patients’ cardiologists, general practitioners, or family doctors.

### 2.5. Data Collection

Data were collected by examining the hospital records. Patient information was recorded in an anonymized Microsoft Office Excel database (Microsoft, Redmond, Washington, DC, USA). Once the data were entered into the database, patient identification was no longer possible.

### 2.6. Inclusion Criteria

The study included adult patients who underwent cardiac surgery with the assistance of extracorporeal circulation (ECC) and were diagnosed with severe heart failure preoperatively. All patients had advanced heart failure, classified as New York Heart Association (NYHA) class III–IV and stage D ACC/AHA (American College of Cardiology/American Heart Association) 2005. Preoperative assessments, such as transthoracic echocardiogram (TTE), transesophageal echocardiogram (TEE), or magnetic resonance imaging (MRI), confirmed a left ventricular ejection fraction (LVEF) of ≤30%. These patients also exhibited clinical signs of decompensation and required therapy with positive inotropic agents.

### 2.7. Exclusion Criteria

Patients with a preoperative LVEF of >30% in TTE, TEE, or MRI were excluded from the study. Additionally, patients who received conservative treatment with medication or non-surgical interventions, such as percutaneous coronary intervention (PCI) or transcatheter aortic valve implantation (TAVI), were also excluded. Patients with acute endocarditis, severe acute or chronic liver disease, SIRS, or sepsis within the two weeks preceding surgery were excluded. Furthermore, patients who were unable to tolerate levosimendan due to excessive vasodilatory effects, as mentioned earlier, were excluded from the study. The study also excluded patients under the age of 18, those who did not consent to the administration of levosimendan, or individuals participating in another study.

### 2.8. Antifibrinolytic Therapy

Antifibrinolytic therapy was routinely administered to all patients. However, aprotinin was not included in our antifibrinolytic regimen. Instead, tranexamic acid (TXA) was the preferred agent and administered at a customized dosage based on the patient’s coagulation profile, as assessed by the anesthesiologists. The use of TXA is an established practice in cardiac surgery to minimize bleeding and the need for transfusions. The dosing of TXA was tailored to each patient, considering factors such as body weight, renal function, and bleeding risk.

### 2.9. Ethics

This study was conducted in compliance with the Declaration of Helsinki and received approval from the Ethics Commission of Rheinland–Paltinate (16 June 2015). As the study was retrospective in nature, the requirement for informed consent was waived.

### 2.10. Measured Parameters

Preoperative evaluation included assessing the LVEF of all patients. Blood chemistry laboratory parameters, including creatinine (mg/dL), creatinine clearance (mL/min), and GFR (mL/min), were used to determine renal function or injury severity. These values were collected preoperatively, upon the patient’s arrival in the ICU, and for the first five days following surgery. The lowest pH and highest lactic acid (mmol/L) values were recorded daily and analyzed to evaluate metabolic changes and postoperative organ perfusion. The C-reactive protein (CRP) (mg/L) levels were measured to assess the extent of the inflammatory response.

### 2.11. Statistical Analysis

Categorical variables were summarized using frequencies and percentages, while continuous variables were presented as the median, interquartile range (IQR), and mean (if normally distributed). The distribution of variables was assessed using the Kolmogorov–Smirnov test. The chi-squared test was employed to evaluate the association of categorical variables. For continuous variables, either the t-test or Mann–Whitney test was used based on their distribution in each cohort. Survival analysis was conducted using the Kaplan–Meier method, and differences in survival between groups were determined using the log-rank test. STATA13 was utilized for statistical analysis. Statistical significance was defined as a *p*-value less than 0.05.

## 3. Results

### 3.1. Demographic Characteristics of the Study Population

The levosimendan group comprised 184 patients (58.59% of the study population), while the control group included 130 patients (41.40%). There were no statistically significant differences observed between the groups in terms of gender (*p* = 0.55) or age (*p* = 0.7). Additionally, no significant differences were found in the body mass index (BMI) or body surface area (BSA), as shown in Table 1.

No significant differences were found between the two groups in terms of the urgency of the surgical intervention (*p* = 0.759) or the complexity of the operation (*p* = 0.09). All patients listed for single surgery underwent CABG, while those who underwent combined surgery received CABG in conjunction with valve surgery or other additional cardiac procedures. Preoperative severe renal failure was more prevalent in the levosimendan group (*p* = 0.012). However, the distribution of patients across the stages of severe renal failure was similar in both groups (*p* = 0.599 for stage 3b, *p* = 0.655 for stage 4, and *p* = 0.856 for stage 5) (Table 2).

### 3.2. Intraoperative Data

The distribution of aortic cross-clamp time significantly differed between the two groups (*p* = 0.041). The control group had a median cross-clamp time of 78 min, while the drug group had a time of 71 min, indicating a longer cross-clamp time in the control group (Table 3).

### 3.3. Impact of Application of Levosimendan on Renal Function and Need for Renal Replacement Therapy

**Figure 1:**
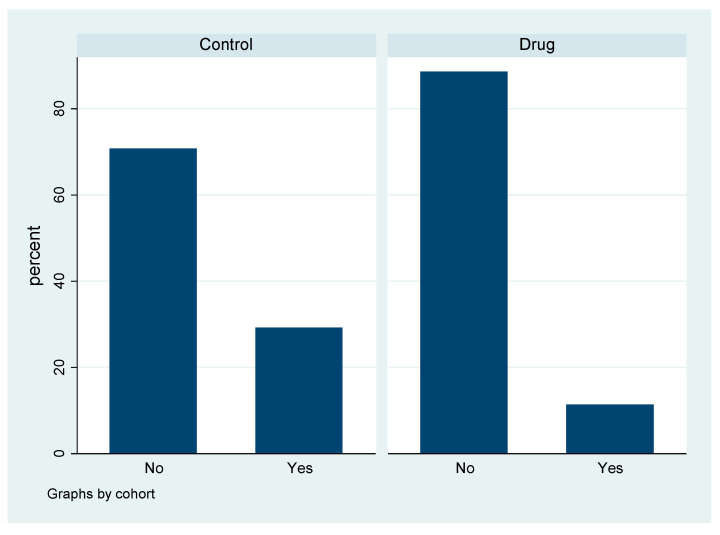
Need for hemodialysis by cohort.

The administration of levosimendan exhibited a clear nephroprotective effect, with a significantly lower need for renal replacement therapy (RRT) after surgery compared with the control group. In the control group, 29.23% of patients required RRT, whereas, in the levosimendan group, only 11.41% required RRT (*p* < 0.001) (Figure 1).

**Figure 2:**
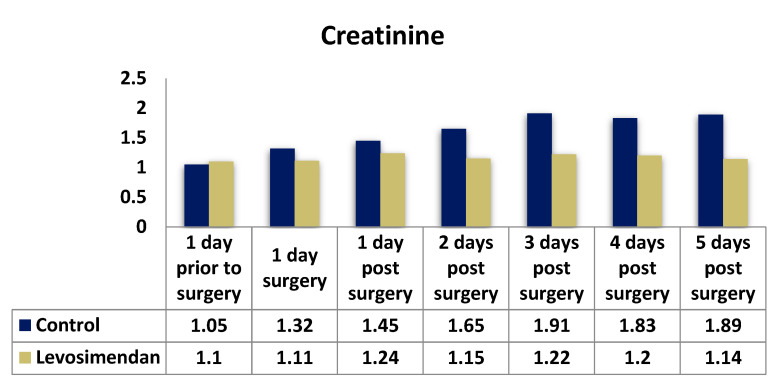
Distribution of creatinine values in the two cohorts.

**Figure 3:**
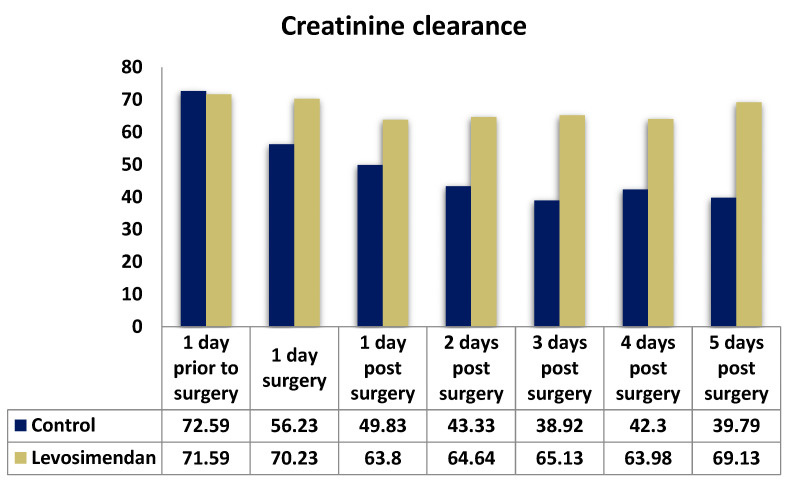
Distribution of creatinine clearance in the two cohorts.

**Figure 4:**
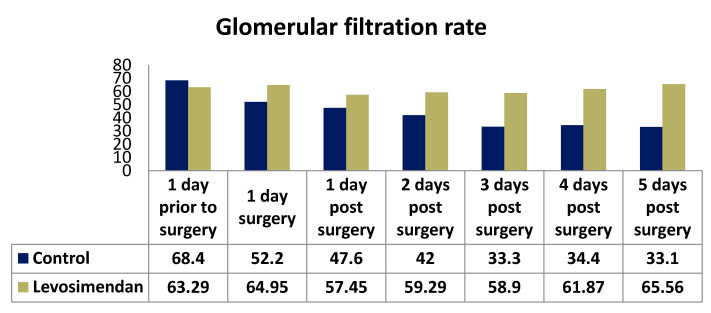
Distribution of GFR in the two cohorts.

Postoperatively, both groups initially exhibited a slight increase in GFR. However, over time, the GFR values showed a regressive trend, approaching or slightly falling below the baseline. Notably, there was a significant difference observed between the two groups in terms of postoperative creatinine and creatinine clearance parameters at all time points. For instance, on the second postoperative day, the median creatinine clearance was 64.64 in the levosimendan group compared with 43.33 in the control group (*p* < 0.001). The median GFR values were consistently higher in the levosimendan group, except for those on the day prior to surgery. Detailed results can be found in Table 4 and Figure 2, Figure 3 and Figure 4.

### 3.4. Other Biochemical Parameters

**Figure 5:**
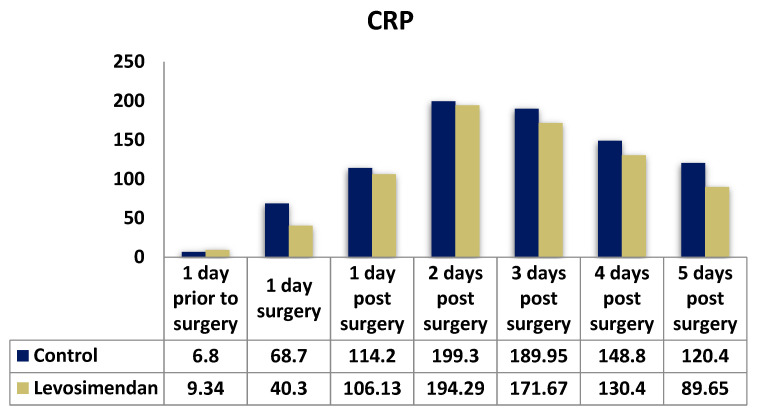
Distribution of CRP values in the two cohorts.

CRP and complement activation play key roles in ischemic myocardial injury [11,12]. The CRP values showed significant differences, starting from day three post-surgery, with lower levels observed in the levosimendan group (Table 5, Figure 5). This implies a reduced inflammatory response in patients treated with levosimendan.

**Figure 6:**
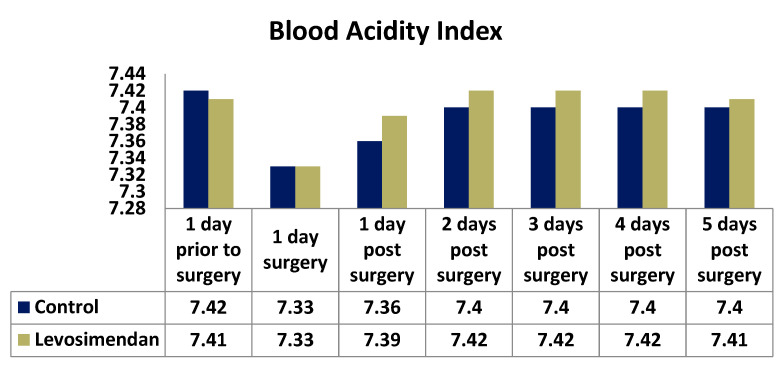
Distribution of pH values in the two cohorts.

The acid–base balance is vital for maintaining the pH of the body’s extracellular fluid (ECF) and ensuring normal physiology and cellular metabolism. The pH levels of both intracellular and extracellular fluids need to be constantly regulated [13]. The distribution of pH differed between the two groups at all time points, with the control group exhibiting lower values (Figure 6).

**Figure 7:**
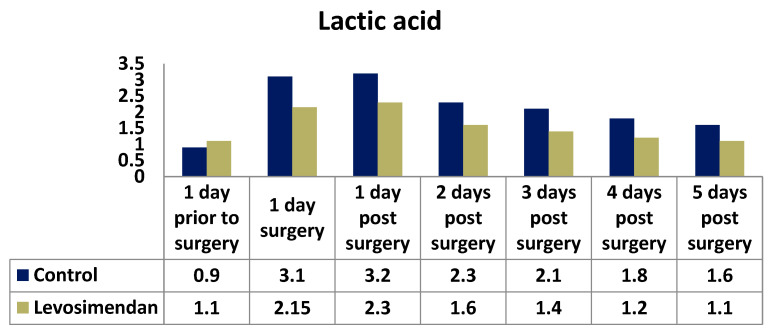
Distribution of lactic acid in the two cohorts.

The distribution of lactic acid significantly varied between the two groups at all time points (Figure 7).

### 3.5. Hemoglobin and Need for Transfusion of Blood Products

Regarding postoperative blood product transfusion, the control group required a significantly higher quantity of red blood cell (RBC) and fresh frozen plasma (FFP) units compared with the levosimendan group (*p* < 0.001 for both parameters). However, there was no observed difference in the need for platelet (PLT) transfusion (*p* = 0.179) (Table 6).

**Figure 8:**
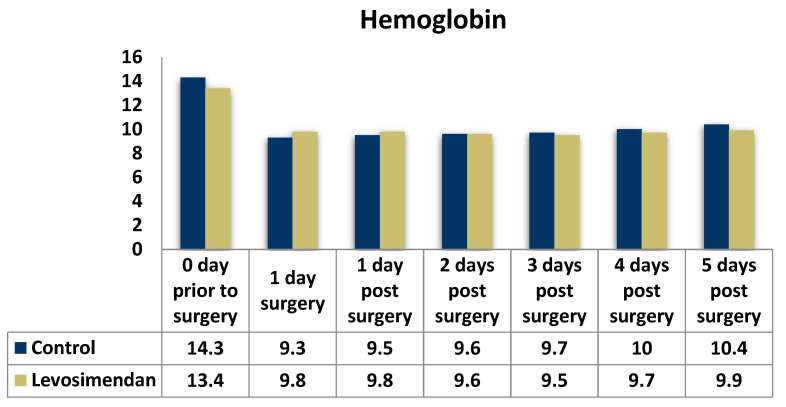
Distribution of the hemoglobin values in the two groups.

The preoperative hemoglobin value was significantly higher in the control group (*p* = 0.003), indicating a higher tendency toward anemia in the levosimendan group. The hemoglobin values on the day of surgery and the first postoperative day appeared to be higher in the levosimendan group (*p* < 0.001 and *p* = 0.047, respectively). However, the only notable disparity in postoperative values was observed on the fifth day, favoring the control group (Table 7, Figure 8).

### 3.6. Other Postoperative Complications

To maintain the integrity of our findings on postoperative renal function and organ perfusion, we implemented measures to minimize potential biases. Therefore, we extensively examined the major postoperative complications in both patient groups, which are detailed in Table 8. No significant differences were observed.

## 4. Discussion

Renal impairment is a common occurrence following cardiac surgery and can be influenced by factors such as impaired cardiac function, preexisting kidney damage, diabetes mellitus, adverse effects of cardiopulmonary bypass (CPB), regional hypoperfusion during extracorporeal circulation, and hemodilution [1]. Our retrospective study found that the perioperative administration of levosimendan exerted a protective effect on renal function, resulting in a reduced need for postoperative RRT. It is important to note that the observed rates of RRT in both groups may differ from those reported in the literature. The higher rate of RRT in the control group could potentially be attributed to the higher proportion of patients with preoperative renal impairment. Notably, both the levosimendan group and the control group included a significant number of patients with preexisting renal dysfunction, which may have contributed to the increased rate of postoperative RRT in both groups. Additionally, the administration of levosimendan was associated with a reduction in the systemic inflammatory response, leading to faster recovery. Levosimendan also improved organ and tissue perfusion, as well as metabolic balance, in the postoperative period. Furthermore, rigorous statistical analysis revealed no significant differences in major postoperative complications between the two groups, reinforcing the validity of our study’s conclusions.

Significant differences were observed between the groups regarding postoperative renal function. The postoperative median GFR values were consistently higher in the levosimendan group (*p* < 0.001). The control group had a significantly higher need for postoperative RRT (*p* < 0.001). It is crucial to consider intraoperative parameters that may influence renal function. While the total duration of the operation and CPB did not differ significantly between the two groups, the control group had a significantly longer cross-clamp time, potentially impacting renal function. Importantly, the levosimendan group had worse baseline characteristics in terms of preoperative renal failure and EuroSCORE, further highlighting the protective effect of levosimendan treatment.

The mechanism underlying the improvement in renal function by levosimendan remains incompletely understood. Levosimendan has demonstrated the ability to increase renal blood flow and decrease renal vascular resistance, leading to improved renal perfusion and oxygenation. Additionally, its nephroprotective effect is believed to stem from its immunomodulatory, anti-inflammatory, and antioxidant properties, which may reduce the release of inflammatory cytokines and subsequent renal injury [14,15,16]. Animal studies have shown that levosimendan effectively mitigates ischemia/reperfusion injury in renal tubules, indicating its potential to enhance renal function [17,18,19,20,21,22]. Bragadottir et al. conducted a study in 2013 that revealed effective vasodilation, particularly in preglomerular vessels, resulting in improved renal blood flow and GFR, without increasing renal oxygen demand. This led to a reduced incidence of AKI and the need for RRT [23]. Similarly, Baysal et al. conducted a prospective, double-blind, randomized study involving 128 participants, reporting significantly better creatinine and GFR values in the levosimendan-treated group, accompanied by a reduced need for RRT [24]. Tholén et al. hypothesized that levosimendan, through its specific effects on renal vasculature, exerts a preferential vasodilating effect on preglomerular resistance vessels, potentially improving renal function in AKI patients without clinical indication for inotropic support. In their single-center, double-blind, randomized controlled trial, they assessed hemodynamically stable adult patients with postoperative AKI within 2 days after cardiac surgery, demonstrating significantly increased renal blood flow (15%) and decreased renal vascular resistance (−18%) with levosimendan compared with the placebo [25].

Lim et al. conducted a meta-analysis of 14 controlled, randomized trials, highlighting a lower incidence of postoperative AKI with levosimendan therapy (7.4% vs. 11.5%) [26]. In contrast, Landoni et al.’s 2010 meta-analysis did not find significant improvements in AKI [27]. A randomized controlled trial by Landoni et al. in 2012 compared levosimendan with a placebo in patients undergoing cardiac surgery with severe left ventricular dysfunction, revealing a significant reduction in AKI incidence, as well as shorter durations of mechanical ventilation and ICU stay [28]. Another study by Tritapepe et al. compared levosimendan with dobutamine in patients undergoing cardiac surgery with impaired LVEF, demonstrating significant improvement in renal function, as measured by creatinine clearance and urine output [29]. Ristikankare et al. in 2012 concluded that levosimendan did not significantly influence kidney function, as measured by specific kidney markers, such as urine N-acetyl-β-glucosaminidase (U-NAG), serum cystatin C, and plasma creatinine [30]. Orriac et al. reported a significantly reduced incidence of kidney failure with postoperative levosimendan administration to cardiac surgery patients with low cardiac output syndrome (LCOS) compared with beta-agonist treatment [31].

A panel of scientists and clinicians from 15 European countries convened in 2013 to reach a consensus on the interpretation of the renal effects of levosimendan described in non-clinical research and clinical study reports. Most reports indicated an improvement in renal function with levosimendan administration in heart failure, sepsis, and cardiac surgery settings, although study designs varied from randomized controlled studies to uncontrolled ones. Notably, the largest heart failure study (REVIVE I and II) did not detect significant changes in renal function [32]. Zhou et al. conducted a meta-analysis in 2015, encompassing 13 trials with 1345 study patients, concluding that levosimendan reduced the incidence of postoperative AKI and the need for RRT [33]. In 2018, Kim et al. performed an arm-based hierarchical Bayesian network meta-analysis involving 95 randomized controlled trials and 28,833 participants, revealing a significant decrease in the rate of postoperative renal dysfunction with levosimendan compared with a placebo [34]. Qiang et al.’s 2018 meta-analysis, which included 25 studies and 3247 patients, demonstrated that levosimendan administration significantly reduced the incidence of postoperative AKI (*p* < 0.0001) and need for RRT (*p* = 0.002) [35]. A recent meta-analysis by Long et al. in 2021 demonstrated that levosimendan may improve renal function in patients with left ventricular dysfunction (LVD). The study, encompassing 28 studies and 5069 patients, showed reductions in the serum creatinine levels and risk of AKI, along with improvements in GFR and urine output. However, no significant reduction in blood urea nitrogen (BUN) was observed [36]. Jawitz et al. aimed to investigate the association of levosimendan administration with postoperative AKI. They stratified patients from the LEVO-CTS trial based on the occurrence and severity of postoperative AKI using the AKIN classification. The study revealed that postoperative AKI is common among high-risk patients undergoing cardiac surgery and is associated with a significantly increased risk of 30-day mortality or the need for renal replacement therapy. However, levosimendan administration was not associated with the risk of postoperative AKI [37]. Zima et al. demonstrated that patients with heart failure who could benefit from levosimendan administration were primarily those with ischemic etiology, well-sustained systemic blood pressure, and concomitant beta-blocker therapy. In comparison with levosimendan, dobutamine was found to be ineffective in patients receiving beta-blocker therapy [7]. Finally, a perspective, multi-center, real-world registry demonstrated that levosimendan infusion increased GFR only in acute heart failure patients with renal dysfunction [38].

It is worth noting that existing meta-analyses encounter significant heterogeneity in the criteria used to diagnose postoperative renal dysfunction (e.g., AKIN criteria, RIFLE criteria, and KDIGO criteria). Consequently, the results of these meta-analyses should be interpreted with caution in clinical practice. The standardization of renal outcome definitions in future studies is necessary to establish more reliable evidence [34].

CPB triggers an SIRS following open-heart surgery, involving compartment activation, cytokine production, and neutrophil sequestration in the lungs, potentially leading to complications. However, the exact mechanism of this complex reaction remains incompletely understood [39]. SIRS during CPB involves the interaction between blood and artificial surfaces, as well as endotoxemia. Protein adsorption initiates the interaction between blood and artificial surfaces, leading to a cascade of chain reactions and the release of various inflammatory mediators, including hormones and autacoids. Consequently, the contact system, coagulation system, complement system, fibrinolysis system, leukocytes, platelets, and endothelial cells are all activated, magnifying the interactions between blood and artificial materials. CPB-associated endotoxemia has been shown to intensify and worsen SIRS during CPB [40].

In our study, the CRP values were regularly assessed as a marker of inflammation. The preoperative CRP values did not significantly differ between the two groups (*p* = 0.158). However, a significant difference was observed immediately after surgery (*p* < 0.001), suggesting a better tolerance of CPB in the levosimendan group. Both groups exhibited a substantial increase in the CRP levels on the second postoperative day, with no significant differences on the first two postoperative days (*p* = 0.555 and *p* = 0.422, respectively). Subsequently, the CRP values declined in both groups. A slight difference was observed on the third postoperative day, favoring the levosimendan group (*p* = 0.047). Significant differences in the CRP values were recorded on the fourth and fifth days (*p* < 0.001), indicating a faster recovery from the inflammatory response in the patients treated with levosimendan.

Our findings are supported by those of previous studies. Adamopoulos et al. and Trikas et al. in 2006 reported a significant anti-inflammatory and anti-apoptotic effect of levosimendan [41,42]. Adamopoulos et al. investigated 96 patients, with one-third receiving levosimendan, dobutamine, or a placebo, and measured pro-inflammatory and apoptotic factors, including interleukin-6 (IL-6), TNF-α, and soluble FAS ligand. Significantly reduced levels of these three parameters were observed only in the levosimendan group [41]. Trikas et al., in a study involving 27 patients, also found a significant and long-lasting effect of levosimendan, leading to reductions in IL-6, soluble FAS ligand, TNF-α, and their corresponding receptors TNF-R_1_ and -R_2_ [42].

Arterial blood gas analysis was performed daily in the ICU to monitor the respiratory and metabolic conditions of each patient. The lowest pH and highest lactic acid values of each day were recorded and analyzed to assess metabolic changes and organ perfusion after surgery. The distribution of pH values differed significantly between the two groups at all time points, indicating a higher tendency toward acidosis in the control group. This finding suggests a potential positive metabolic effect of levosimendan, reducing the degree of postoperative metabolic acidosis.

The lactic acid values were used as markers of circulatory function and tissue perfusion, providing insights into the adequacy of cardiac output and oxygen supply to tissues. The distribution of lactic acid values differed significantly between the two groups at all time points during the first five postoperative days. Fang et al. conducted a similar study involving 36 patients requiring intensive care due to severe sepsis. They observed lower measured lactate values in the levosimendan-treated group, accompanied by improvements in hemodynamic status [43]. Another recent study by Xu et al. demonstrated that levosimendan, compared with dobutamine, significantly reduced the blood lactic acid at 24 h in patients with septic myocardial contractility impairment [44].

To sum up, our results support the potential positive inotropic effect of levosimendan and its metabolites, as well as their protective role against postoperative LCOS. It is important to acknowledge the retrospective nature of this study and the possible biases associated with such designs. Therefore, comparisons with the existing literature, especially randomized controlled trials, should be interpreted cautiously, considering all limitations.

## 5. Limitations

This retrospective study was conducted at a single center and included patients undergoing various cardiac operations. Therefore, caution should be exercised when interpreting our findings, and definitive conclusions should not be drawn solely based on this study. However, our results are derived from real-life data reflecting routine clinical practice. To establish the precise value of perioperative levosimendan administration, prospective randomized controlled trials are warranted.

Another potential bias to consider is the impact of cardiac surgery on cardiac output and renal function. Successful cardiac surgery can improve cardiac output, which may subsequently contribute to better postoperative renal function. In our study, both the levosimendan group and the control group underwent similar surgical procedures for their underlying cardiac conditions. These procedures were performed at the same institution, under comparable conditions, and by the same group of surgeons. Therefore, any potential benefits resulting from the surgical intervention itself, such as improved cardiac output, would have been similar between the two groups.

Furthermore, it is important to acknowledge that our institution serves as a local reference center for high-risk patients with severe comorbidities. These patients often require complex surgical procedures and may have a higher predisposition for postoperative complications, including renal dysfunction, compared with the general population reported in the literature. The higher rate of RRT observed in our study may reflect the unique characteristics of our patient population and the specific context of our center.

Additionally, it is worth noting that pH values, while being reliable indicators of a patient’s metabolic condition, can be influenced by other factors, such as metabolic or respiratory acidosis, ventilator settings, the use of non-invasive ventilation after extubation, and glycemic status. These factors should be taken into consideration when interpreting pH values.

## 6. Conclusions

Renal dysfunction is a prevalent and significant complication following cardiac surgery, contributing to increased morbidity and mortality rates. The administration of levosimendan in cardiac surgery patients with severe heart failure exhibited improvements in renal function and a reduction in the incidence of acute kidney injury. Moreover, levosimendan was associated with a decrease in the inflammatory response and enhancement of tissue and organ perfusion. While the precise mechanism of action remains incompletely understood, the vasodilatory and anti-inflammatory properties of levosimendan are believed to contribute to these beneficial effects. Nevertheless, further research is warranted to validate these findings, ascertain the optimal dosage and timing of levosimendan administration, and explore any potential adverse effects associated with its use in cardiac surgery patients. Consequently, we advocate for the inclusion of levosimendan as an adjunctive therapy in complex cardiac surgical interventions, particularly for patients with severe heart failure. However, to advance our understanding in this field and investigate the potential drawbacks of this treatment, additional prospective randomized trials are needed.

## Figures and Tables

**Table 1:** Patients’ characteristics at baseline.

	Control (N: 130)	Levosimendan (N: 184)	*p*-Value
**Gender (N, %)**			0.551
Males	106 (81.5)	145 (78.8)	
Females	24 (18.5)	39 (21.2)	
**Age (years)**			
Median (IQR)	71 (63, 76)	69 (63, 76.5)	0.703
**Body mass index (BMI)**			
Median (IQR)	26.8 (25.4, 28.7)	27.6 (25.5, 30.4)	0.072
**Body surface area (BSA)**			
Median (IQR)	2.0 (1.9, 2.1)	2.0 (1.9, 2.1)	0.096

**Table 2:** Patients’ baseline clinical characteristics prior to surgery.

	Control (N: 130)	Levosimendan (N: 184)	*p*-Value
**Euroscore 1**			
Median (IQR)	15.7 (8.1, 27.9)	22.8 (11.6, 42.5)	**<0.001**
**Euroscore 2**			
Median (IQR)	5.4 (3.4, 10.5)	8.8 (5.1, 17.1)	**<0.001**
**Type of surgery (N, %)**			
Elective/regular surgery	96 (73.9)	133 (72.3)	0.759
Emergency surgery	34 (26.2)	51 (27.7)	
**Simple/combination surgery (N, %)**			
Simple surgery	95 (73.1)	118 (64.1)	0.095
Combination surgery	35 (26.9)	66 (35.9)	
**Severe renal failure (GFR < 45 mL/min/1.73m^2^) (N, %)**			
No	112 (86.2)	137 (74.5)	**0.012**
Yes	18 (13.9)	47 (25.5)	
Stage 3b	12 (66.7)	28 (59.6)	0.599
Stage 4	4 (22.2)	13 (27.7)	0.655
Stage 5	2 (11.1)	6 (12.8)	0.856
**Ejection fraction (EF)**			
Median (IQR)	30 (25, 30)	20 (18.5, 30)	**<0.001**

**Table 3:** The intraoperative data, including the total duration of the operation, the cross-clamping time, and the duration of the cardiopulmonary bypass.

	Control (N: 130)	Levosimendan (N: 184)	*p*-Value
**Duration of surgery (min)**			
Median (IQR)	218.0 (177.5, 262.8)	227.5 (184.0, 283.8)	0.220
**Bypass time (min)**			
Median (IQR)	113.0 (91.0, 154.0)	111.0 (82.0, 153.3)	0.313
**Aortic cross-clamp time (min)**			
Median (IQR)	78.0 (56.8, 103.3)	71.0 (54.0, 92.0)	**0.041**

**Table 4:** Renal function parameters at different time points in the two cohorts.

	Control (N: 130)	Levosimendan (N: 184)	*p*-Value
**Creatinine (mg/dL); median (IQR)**			
0 days prior to surgery	1.1 (0.8, 1.3)	1.1 (0.9,1.5)	**0.050**
1 day of surgery	1.3 (1.0, 1.7)	1.1 (0.9, 1.5)	**0.002**
2 1st postop. day	1.5 (1.1, 1.9)	1.2 (0.9, 1.8)	**0.005**
3 2nd postop. day	1.7 (1.1, 2.8)	1.2 (0.8, 2.0)	**<0.001**
4 3rd postop. day	1.9 (1.1, 3.4)	1.2 (0.8, 2.1)	**<0.001**
5 4th postop. day	1.8 (1.1, 3.5)	1.2 (0.8, 2.2)	**<0.001**
6 5th postop. day	1.9 (1.2, 3.3)	1.1 (0.8, 1.9)	**<0.001**
**Creatinine clearance (mL/min); median (IQR)**			
0 days prior to surgery	72.6 (54.6, 91.3)	71.6 (48.0, 100.6)	0.506
1 day of surgery	56.2 (43.3, 76.5)	70.2 (47.4, 102.2)	**0.001**
2 1st postop. day	49.8 (40.3, 69.8)	63.8 (44.4, 97.1)	**0.002**
3 2nd postop. day	43.3 (26.5, 73.3)	64.6 (35.6, 106.7)	**<0.001**
4 3rd postop. day	38.9 (20.7, 65.8)	65.1 (36.5, 102.8)	**<0.001**
5 4th postop. day	42.3 (19.4, 69.2)	64.0 (32.2, 107.8)	**<0.001**
6 5th postop. day	39.8 (20.9, 66.4)	69.1 (37.0, 104.6)	**<0.001**
**Glomerular filtration rate (GFR) (mL/min); median (IQR)**			
0 days prior to surgery	68.4 (55.0, 82.3)	63.3 (46, 83.0)	0.061
1 day of surgery	52.2 (38.9, 72.0)	65.0 (43.7, 86.3)	**0.002**
2 1st postop. day	47.6 (33.2, 66.7)	57.5 (38.3, 83.6)	**0.004**
3 2nd postop. day	42.0 (20.8, 66.4)	59.3 (32.5, 90.6)	**<0.001**
4 3rd postop. day	33.3 (15.5, 63.2)	58.9 (29.8, 88.0)	**<0.001**
5 4th postop. day	34.4 (15.2, 63.0)	61.9 (28.6, 91.7)	**<0.001**
6 5th postop. day	33.1 (18.1, 65.7)	65.6 (35.7, 89.9)	**<0.001**

**Table 5:** CRP, pH, and lactic acid values at different time points in the two cohorts.

	Control (N: 130)	Levosimendan (N: 184)	*p* Value
**C Reactive Protein (CRP) (mg/L); median (IQR)**			
0 days prior to surgery	6.8 (3.6, 17.3)	9.3 (4.0, 22.3)	0.158
1 day of surgery	68.7 (34.7, 88.1)	40.3 (22.7, 72.7)	**<0.001**
2 1st postop. day	114.2 (78.4, 156.3)	106.1 (56.1, 186.3)	0.555
3 2nd postop. day	199.3 (159.1, 243.8)	194.3 (151.8, 239.9)	0.422
4 3rd postop. day	190.0 (147.2, 273.9)	171.7 (133.9, 227.8)	**0.047**
5 4th postop. day	148.8 (109.9, 236.0)	130.4 (90.3, 173.0)	**<0.001**
6 5th postop. day	120.4 (76.0, 192.7)	89.7 (55.5, 131.2)	**<0.001**
**Blood pH; median (IQR)**			
0 days prior to surgery	7.42 (7.41, 7.43)	7.41 (7.39, 7.43)	**<0.001**
1 day of surgery	7.33 (7.27, 7.37)	7.33 (7.29, 7.36)	0.942
2 1st postop. day	7.36 (7.32, 7.40)	7.39 (7.36, 7.42)	**<0.001**
3 2nd postop. day	7.40 (7.36, 7.43)	7.42 (7.39, 7.44)	**0.001**
4 3rd postop. day	7.40 (7.36, 7.43)	7.42 (7.39, 7.44)	**0.003**
5 4th postop. day	7.40 (7.37, 7.42)	7.42 (7.39, 7.44)	**0.002**
6 5th postop. day	7.40 (7.38, 7.42)	7.41 (7.40, 7.43)	**0.001**
**Lactic acid (mmol/L); median (IQR)**			
0 days prior to surgery	0.9 (0.7, 1.2)	1.1 (0.9, 1.3)	**<0.001**
1 day of surgery	3.1 (2.0, 6.2)	2.2 (1.4, 3.9)	**<0.001**
2 1st postop. day	3.2 (2.1, 5.1)	2.3 (1.6, 4.3)	**0.001**
3 2nd postop. day	2.3 (1.6, 3.2)	1.6 (1.2, 2.4)	**<0.001**
4 3rd postop. day	2.1 (1.4, 3.2)	1.4 (1.0, 2.0)	**<0.001**
5 4th postop. day	1.8 (1.2, 2.4)	1.2 (1.0, 1.7)	**<0.001**
6 5th postop. day	1.6 (1.2, 2.2)	1.1 (0.9, 1.5)	**<0.001**

**Table 6:** Recorded need for transfusion of RBCs, FFPs, and PLTs.

	Control (N: 130)	Levosimendan (N: 184)	*p*-Value
**Units of red blood cells (RBC) transfused**			
Median (IQR)	8.0 (4.0, 12.0)	4.0 (2.0, 11.5)	**<0.001**
**Fresh-frozen plasma (FFP) units transfused**			
Median (IQR)	8.0 (4.0, 12.0)	4.0 (0.0, 8.0)	**<0.001**
**Total platelet (PLT) units transfused**			
Median (IQR)	0.0 (0.0, 2.0)	0.0 (0.0, 2.0)	0.179

**Table 7:** The recorded values of hemoglobin during the first 5 postoperative days.

	Control (N: 130)	Levosimendan (N: 184)	*p*-Value
**Hemoglobin (g/dL); median (IQR)**			
0 days prior to surgery	14.3 (12.5, 15.4)	13.4 (11.7, 15.0)	**0.003**
1 day of surgery	9.3 (8.6, 10.0)	9.8 (9.9, 10.7)	<0.001
2 1st postop. day	9.5 (8.6, 10.5)	9.8 (8.9, 10.8)	0.047
3 2nd postop. day	9.6 (8.9, 10.4)	9.6 (8.9, 10.4)	0.956
4 3rd postop. day	9.7 (9.0, 10.4)	9.5 (8.8, 10.3)	0.315
5 4th postop. day	10.0 (9.2, 10.8)	9.7 (8.9, 10.7)	0.082
6 5th postop. day	10.4 (9.4, 11.3)	9.9 (9.1, 10.9)	**0.028**

**Table 8:** The most important recorded postoperative complications for both cohorts.

Complications (N, %)	Control (N: 130)	Levosimendan (N: 184)	*p*-Value
Major bleeding requiring surgical revision	3 (2.3)	4 (2.2)	<0.999
Stroke	2 (1.5)	3 (1.6)	<0.999
Pneumonia	7 (5.4)	9 (4.9)	0.845
Urinary tract infection	8 (6.2)	11 (6.0)	0.949
Wound infection	3 (2.3)	4 (2.2)	<0.999
Mediastinitis	1 (0.8)	1 (0.5)	<0.999
Sternal instability	3 (2.3)	4 (2.2)	<0.999
Deep venous thrombosis	2 (1.5)	2 (1.1)	<0.999
Sepsis	5 (3.9)	7 (3.8)	0.985
Multiple organ dysfunction	4 (3.0)	5 (2.7)	0.851

## Data Availability

The data presented in this study are available upon request from the corresponding author.
